# Is FDG-PET/CT Scan Useful in the Detection of Subcentimeter Mediastinal Lymph Node Involvement in Patients With Lung Carcinoma?

**DOI:** 10.7759/cureus.74572

**Published:** 2024-11-27

**Authors:** Muhammad Omer Altaf, Hamd Zahra, Hira Majied, Imran Khalid Niazi, Hira Farooq

**Affiliations:** 1 Department of Radiology, Shaukat Khanum Memorial Cancer Hospital and Research Centre, Lahore, PAK; 2 Department of Radiology, Shalamar Hospital, Shalamar Medical and Dental College, Lahore, PAK; 3 Department of Radiology, University Hospitals of North Midlands NHS Trust, Stoke-on-Trent, GBR

**Keywords:** fdg-pet/ct, lung carcinoma, lymph nodes, nodal metastasis, subcentimeter

## Abstract

Background

Early staging of lung carcinoma (CA) is pivotal in planning the treatment. Lymph node metastasis can be detected by imaging and invasive procedures. The 18F-fluorodeoxyglucose positron emission tomography/computed tomography (FDG-PET/CT) is an emerging noninvasive imaging modality in detecting nodal metastasis.

Objective

This study aimed to determine the usefulness of the FDG-PET/CT scan in detecting subcentimeter mediastinal lymph node involvement in lung CA patients by taking histopathology as the gold standard.

Materials and methods

We conducted a retrospective cross-sectional study at a tertiary care cancer hospital in Pakistan over four and half years, from January 2015 to June 2019. All patients suffering from non-small cell lung CA (NSCLC) having avid subcentimeter nodes on FDG-PET/CT were included. The findings obtained on FDG-PET/CT were correlated with histopathological findings after endobronchial ultrasound (EBUS). The results were formulated using IBM SPSS Statistics for Windows, Version 21 (Released 2012; IBM Corp., Armonk, New York, United States).

Results

Results showed that 42/380 total lung CA patients had avid subcentimeter lymph nodes obtained on FDG-PET/CT. A total of 22/42 (52.4%) lymph nodes appeared to be benign, and 20/42 (47.6%) lesions were malignant on histopathology. FDG-PET/CT sensitivity is calculated to be 95%, specificity is 68%, positive predictive value is 73%, negative predictive value is 94%, and accuracy is 80.9%. Using the receiver operating characteristic (ROC) curve, sensitivity and specificity were seen in nodes of size 7.5 mm and the maximum standardized uptake value (SUV max) of 5.5 as cutoff values as manifested by area under the curve (AUC).

Conclusion

FDG-PET/CT was proven to have high sensitivity and accuracy but a low specificity rate to detect nodal involvement in lung CA patients. The high false-positive rates are mainly due to increased prevalence of endemic lung infectious disease.

## Introduction

Lung carcinoma (CA) is the most common CA worldwide constituting 13% of all the diagnosed cancer cases [1]. The highest morbidity and mortality rate associated with lung CA demand for its early diagnosis and treatment [1,2]. In addition to this, lung cancer is classified into four main types as seen under a light microscope: adenocarcinoma, squamous cell CA, large cell CA, and small cell CA. Smoking is a key factor in causing all these types [3], as it is believed that the substances in cigarette smoke are responsible for causing continuous DNA damage which ultimately leads to genetic mutations seen in lung cancer [4].

The assessment of mediastinal lymphadenopathy holds significant importance in lung cancer staging [1]. Surgical intervention is favored when lymph nodes remain unaffected, while radiotherapy and chemotherapy become the preferred treatment modalities if lymph node involvement is detected. Therefore, evaluating lymphadenopathy plays a crucial role in both diagnosis and prognosis [5]. 

In 2007, the European Society of Thoracic Surgeons (ESTS) introduced an algorithm to identify mediastinal lymphadenopathy in non-small cell lung CA (NSCLC), combining imaging, surgical, and endoscopic techniques. However, these surgical and endoscopic methods were invasive and carried risks of associated complications [6]. Computed tomography (CT) scans and magnetic resonance imaging (MRI) are standard imaging modalities for evaluating mediastinal lymph node status, but they have limitations. CT scans offer low sensitivity and specificity of 64% and 62%, respectively [7], while MRI may fail to detect calcification in the lymph nodes and can misinterpret a group of discrete nodes as a single large one due to poor spatial resolution. Moreover, their efficacy in detecting nodes measuring 1 cm or less remains unestablished. Furthermore, both CT and MRI often miss microinvasions and metastatic lesions [8].

Another noninvasive imaging technology introduced lately is 18F-fluorodeoxyglucose positron emission tomography (FDG-PET). It has a sensitivity of 84% and a specificity of 89% in assessing mediastinal lymph node pathology [9]. Recent studies have shown that PET and CT, when combined, can provide higher diagnostic accuracy by overcoming the limitations of both PET and CT [10].

According to the American Joint Committee on Cancer (AJCC) and the National Comprehensive Cancer Network (NCCN) guidelines for imaging, in case of CA lung suspicion, a chest X-ray is the first imaging modality to be done. Guidelines recommend a contrast-enhanced CT (CECT) scan of the chest and upper abdomen (including the adrenal glands) for further evaluation. In patients who are eligible for curative treatment in addition to having no signs of metastasis on CT scan, an FDG-PET/CT is suggested [11].

The significance of FDG-PET/CT scan in identifying subcentimeter mediastinal lymphadenopathy in NSCLC patients, taking histopathology as a gold standard, will be discussed in this study. This study will focus mainly on the presence of metastatic subcentimeter lymph nodes depending upon their size and metabolic activity This will aid in sparing patients from unnecessary invasive diagnostic procedures that could potentially result in various complications.

## Materials and methods

It is a retrospective cross-sectional study conducted at a tertiary care cancer center, Shaukat Khanum Memorial Cancer Hospital and Research Centre, Lahore, in collaboration with its Radiology and Pathology Department. The study duration was from January 1, 2015, to June 30, 2019.

Following approval from the Institutional Review Board (IRB), we retrospectively analyzed data of patients diagnosed with NSCLC via histopathology between January 1, 2015, and June 30, 2019. Inclusion and exclusion criteria were applied as described ahead. Selection of patients who exhibited metabolically active subcentimeter mediastinal lymph nodes on FDG-PET/CT scan was done. Patients of both sexes, male and female, between 16 and 75 years of age were included. Patients from diverse socioeconomic backgrounds were a part of our study. The socioeconomic status of patients showed variability, including patients from the poor, middle class, and upper class. Additionally, a notable majority of the patients who were included in this study had a history of smoking, with relatively fewer patients with no smoking history. The patients who were left out of this study were the ones with evidence of distant metastases on CT scan or PET done prior to surgical intervention as well as those who underwent FDG-PET/CT scan within three months before endobronchial nodal biopsy. In addition to the preceding criteria, cases with incomplete patient records stored in the hospital information system were excluded.

Patients, included in the study, had their presenting complaints, complete imaging investigations, and prior medical and treatment histories thoroughly reviewed. Complete confidentiality of all records was not overlooked. Detailed cancer staging prior to surgical intervention was ensured in all patients included in the study. Each patient underwent whole-body FDG-PET with an integrated CT scan. In order to reduce observational bias, these scans in each case were then subjected to careful evaluation using reconstructed and reformatted axial, coronal, and sagittal views by two expert radiologists independently, each with over five years of experience in the field.

To classify a lymph node as active it was established that it exhibits higher metabolic activity in comparison with the background hepatic activity, in each case. Lymph nodes fulfilling either one or both of the two criteria mentioned in the following sentence were marked malignant. Nodes with metabolic activity exceeding 4 to 4.5 SUV and/or a size greater than 7 to 7.5 mm FDG-PET/CT, based on individual assessments by two independent radiologists.

The body mass index of each subject, time of administration, and administrative activity of 18 FDG at the time of the test were noted to calculate the maximum standardized uptake value (SUV max). Cytopathological staging of each patient on endobronchial ultrasound (EBUS)-guided biopsy was done, and results were categorized into benign or malignant nature. 

Data analysis was done using IBM SPSS Statistics for Windows, Version 21 (Released 2012; IBM Corp., Armonk, New York, United States), and inferential tests were applied to describe frequencies and distributions by age, gender, size, and avidity. Sensitivity and specificity were calculated, followed by multivariate analysis and correlations using Pearson’s chi-square analysis. A receiver operating characteristic (ROC) curve was constructed to determine SUV max accuracy via the area under the curve (AUC). The results were obtained and correlated using chi-square analysis, keeping the p-value of <0.05 as significant.

## Results

The total number of patients with lung CA who presented to our department from January 1, 2015, to June 30, 2019, were 380. Out of the total number of patients, 253 (66.5%) had nodal disease with mediastinal nodal size greater than 1 cm in size at the time of acquisition of PET-CT, while 127 (33.4%) patients had lymph nodes less than 1 cm. Around 85 (22.3%) out of 127 patients had shown no increased activity in the lymph nodes in comparison with the background hepatic metabolic activity which was taken as a control. The remaining 42 (11% of n) out of 127 patients having lung carcinoma had subcentimeter avid lymph nodes; hence, they were included in our study population as per inclusion criteria. These 42 patients, selected to be included in our study, displayed a diverse demographic profile (Tables 1-4). The mean age of patients was 68 years (age range: 53-84 years) with a standard deviation of 8.142, with the most common age group being 66-70 years (eight patients). Closely following were individuals aged 61-65, 71-75, 76-80, and 81-85 years, each group consisting of seven patients. The lowest number of patients was observed in the 56-60 years age group, with only six patients (Table 1). Of all the patients included in our study, only 10 (23.8% of n) were females, and the rest (32, 76.2% of n) were males, with a male-to-female ratio of 3.2: 1 (Table 2). In addition to this, patients of different socioeconomic statuses were a part of our study group. Out of the 42 total patients, 18 (42.8%) belonged to a poor socioeconomic background, 18 (42.8%) were middle class, and six (14.3%) were classified to be from upper socioeconomic status (Table 3). Furthermore, a notable majority, 27 (64.2%) patients, had a history of smoking, while 15 (35.7%) patients did not smoke (Table 4). Among the ones who did not have a history of smoking, the majority were females.

**Table 1:** Distribution of patients in the study across various age groups

Age group	Number of patients
56-60	6
61-65	7
66-70	8
71-75	7
76-80	7
81-85	7

**Table 2:** Gender distribution of patients in the study

Male	Female
76.2%	23.8%

**Table 3:** Distribution of patients in the study by socioeconomic status

Socioeconomic status	Number of patients
Poor	18
Middle class	18
Upper class	06

**Table 4:** Patient distribution based on smoking history in the study

Smoking history	Number of patients
Present	27
Not present	15

Supported by histopathological evaluation, out of 42 patients, 25 (59.5%) were diagnosed to have adenocarcinoma, while 17 (40.5%) were diagnosed with squamous cell carcinoma. It was revealed that most of the patients with adenocarcinoma presented at stage II B, whereas those with squamous cell carcinoma presented at stage III C. No other types of carcinoma were observed among patients selected for this study. Out of the total 42 patients, 22 (52.4%) had lymph nodes that were classified as benign. Among 22 patients with lymph nodes labeled as benign, six (14.3%) had lymph nodes showing signs of inflammation, and 16 (38.1%) displayed reactivity. On the other hand, histopathological analysis confirmed malignancy in 20 (47.6%) lesions.

FDG-PET/CT scan findings showed that sizes of lymph nodes ranged from 5 to 9 mm. Out of the total of 42 patients, 32 (76.2% of the total) had lymph node sizes between 6 and 8 mm (with a mean of 7.1 mm and a standard deviation of 1.284). SUV values, pertaining to metabolic activity, ranged from 2 to 9 (with a mean metabolic activity of 4.93 SUV and a standard deviation of 1.813). The relationship between size and metabolic activity along with the benign and malignant nature of lymph nodes as revealed on histopathology is given in Tables 5-6, respectively. In this study’s findings, larger size and high metabolic activity were the factors that were observed to have an association with the presence of malignant disease, indicating a statistically significant correlation (p-value < 0.05).

**Table 5:** Depiction of individual evaluation of pathology results for nodal sizes and metabolic activities FDG-PET/CT: 18F-fluorodeoxyglucose positron emission tomography/computed tomography; SUV: standardized uptake value

FDG-PET/CT variables	mm/SUV	Pathology decision				
		Benign		Malignant		
		Count	Table N %	Count	Table N %	p-value
Mediastinal node size in millimeters	5	2	4.80%	2	4.80%	0.033
	6	11	26.20%	3	7.10%	
	7	2	4.80%	2	4.80%	
	8	7	16.70%	7	16.70%	
	9	0	0.00%	6	14.30%	
Mediastinal node avidity SUV	2	2	4.80%	0	0.00%	0.024
	3	6	14.30%	1	2.40%	
	4	9	21.40%	4	9.50%	
	5	3	7.10%	2	4.80%	
	6	1	2.40%	5	11.90%	
	7	1	2.40%	4	9.50%	
	8	0	0.00%	2	4.80%	
	9	0	0.00%	2	4.80%	

**Table 6:** Depiction of combined evaluation of pathology results for nodal sizes and metabolic activities FDG-PET/CT: 18F-fluorodeoxyglucose positron emission tomography/computed tomography; SUV: standardized uptake value

FDG-PET/CT variable		Pathology decision			
Mediastinal node avidity SUV	Mediastinal node size in millimeters	Benign		Malignant	
		Count	Table N %	Count	Table N %
2	6	2	4.80%	0	0.00%
3	6	4	9.50%	0	0.00%
	7	2	4.80%	0	0.00%
	8	0	0.00%	1	2.40%
4	5	2	4.80%	0	0.00%
	6	2	4.80%	0	0.00%
	7	0	0.00%	2	4.80%
	8	5	11.90%	1	2.40%
	9	0	0.00%	1	2.40%
5	6	2	4.80%	2	4.80%
	8	1	2.40%	0	0.00%
6	5	0	0.00%	1	2.40%
	6	1	2.40%	1	2.40%
	8	0	0.00%	2	4.80%
	9	0	0.00%	1	2.40%
7	5	0	0.00%	1	2.40%
	8	1	2.40%	0	0.00%
	9	0	0.00%	3	7.10%
8	8	0	0.00%	1	2.40%
	9	0	0.00%	1	2.40%
9	8	0	0.00%	2	4.80%

Multivariate analysis revealed that nodal size and SUV max are predictors of nodal metastasis, with OD of 1.925 (95% CI: 1.115-3.323, p = 0.033) and OD of 2.667 (95% CI: 1.467-4.847, p = 0.024), respectively.

**Figure 1:**
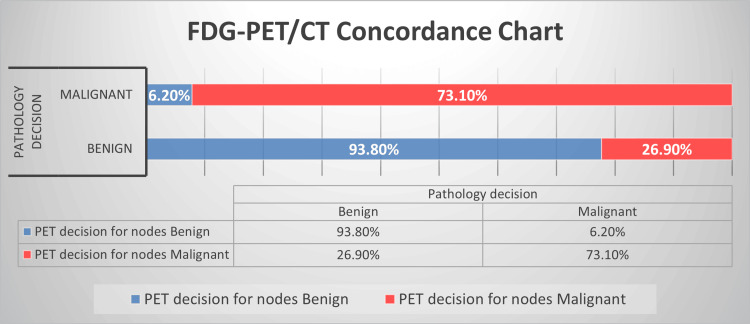
Depiction of FDG-PET/CT concordance with pathology result for subcentimeter nodal involvement in lung carcinoma patients FDG-PET/CT: 18F-fluorodeoxyglucose positron emission tomography/computed tomography

The concordance between decision taken on FDG-PET/CT and histopathology decision was calculated. It was observed that 15/16(93.8%) patients with benign nodes and 19/26 (73%) patients with malignant nodes labeled on FDG-PET/CT were in concordance with the results on pathology as shown in Figure 1.

**Figure 2:**
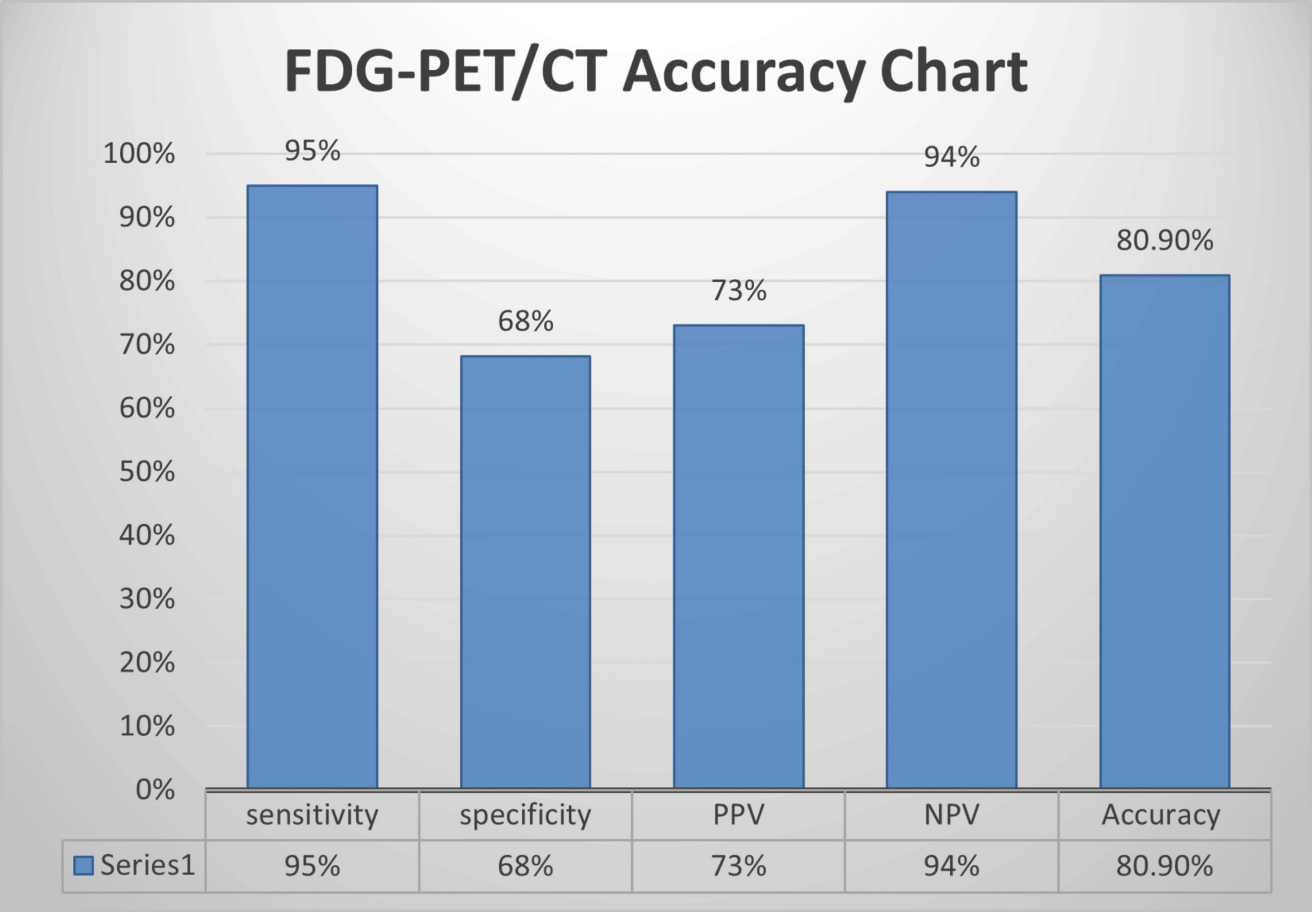
FDG-PET/CT accuracy chart depicting calculated specificity and sensitivity rates based on the results of the study FDG-PET/CT: 18F-fluorodeoxyglucose positron emission tomography/computed tomography

Taking into account the size and metabolic activity for benign and malignant lesions on FDG-PET/CT as mentioned in the methodology section, the sensitivity of FDG-PET/CT is 95%, specificity is 68%, positive predictive value is 73%, negative predictive value is 94%, and accuracy is calculated to be 80.9% as shown in Figure 2. It was obtained after taking the mean of the findings by two experienced radiologists, with a good inter-rater agreement (k value: 0.854).

**Figure 3:**
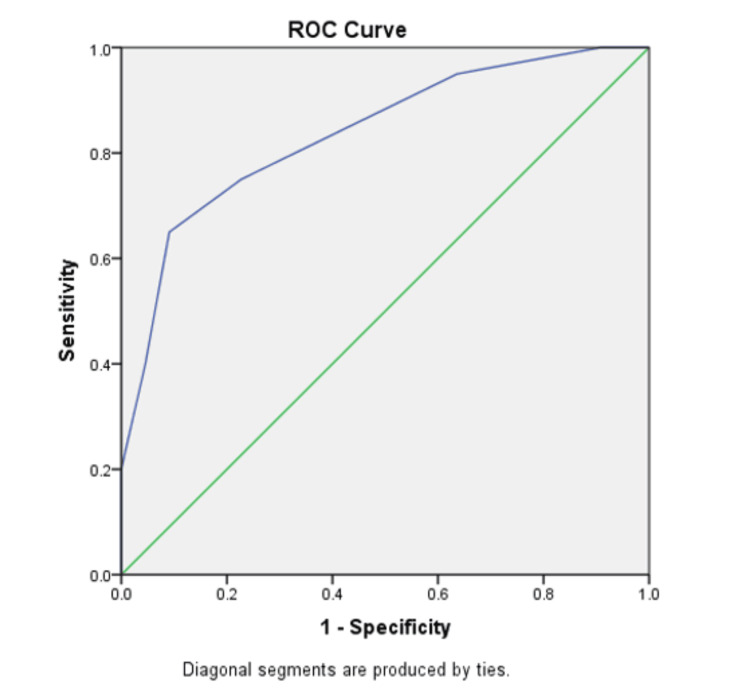
ROC curve depicting graphical plot illustration for the diagnostic ability of optimal nodal metabolic activity (SUV max) SUV max: maximum standardized uptake value; ROC: receiver operating characteristic Area under the curve = 0.837; standard error = 0.062

**Figure 4:**
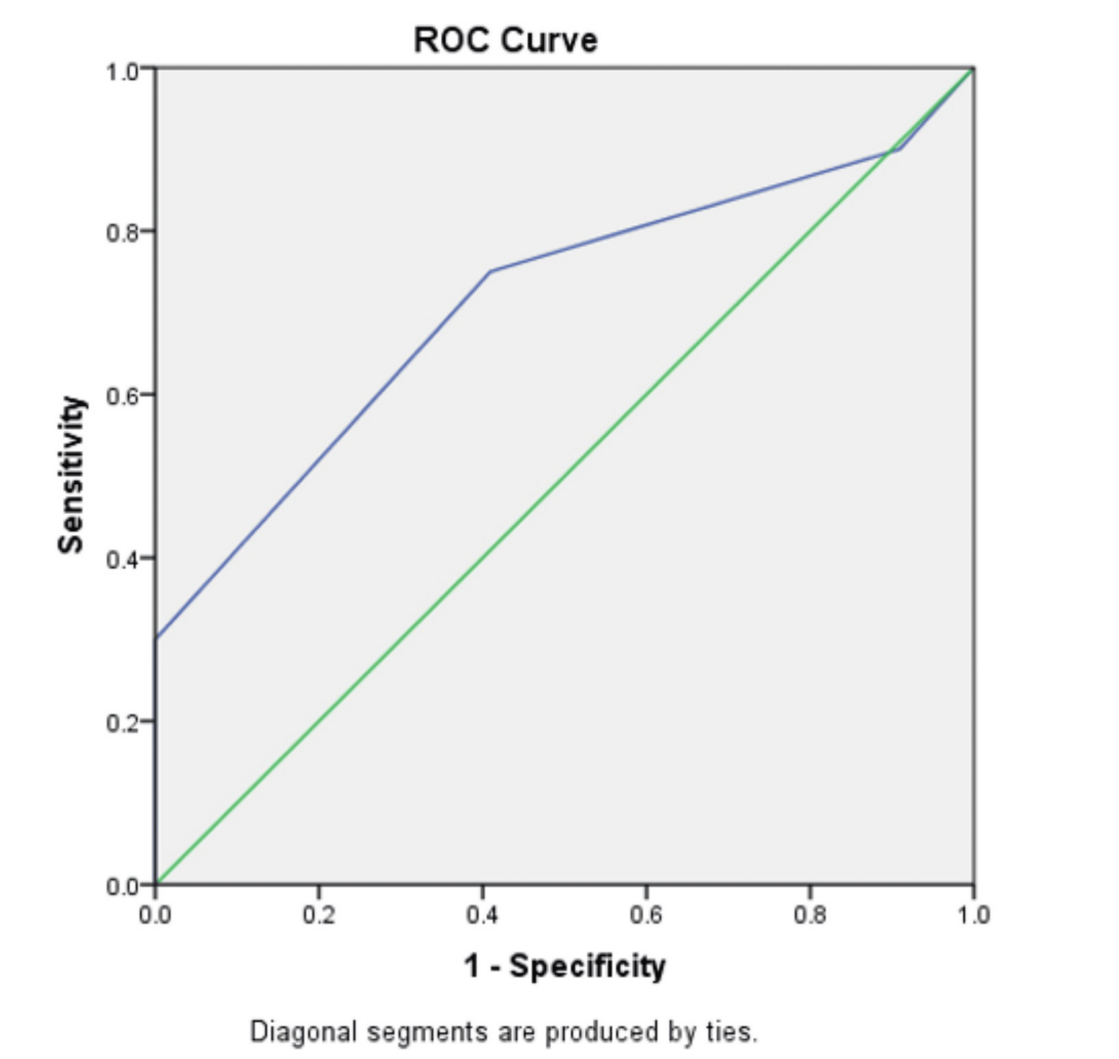
ROC curve depicting graphical plot illustration for the diagnostic ability of optimal nodal size (mm) ROC: receiver operating characteristic Area under the curve = 0.714; standard error = 0.083

ROC curves were designed to determine the optimal cutoff values for size and SUV max to maximize the sensitivity and specificity of FDG-PET/CT. It was observed that the maximum sensitivity and specificity values were seen in nodes of size 7.5 mm and SUV max of 5.5 as cutoff values as manifested by the AUC in Figures 3-4.

**Figure 5:**
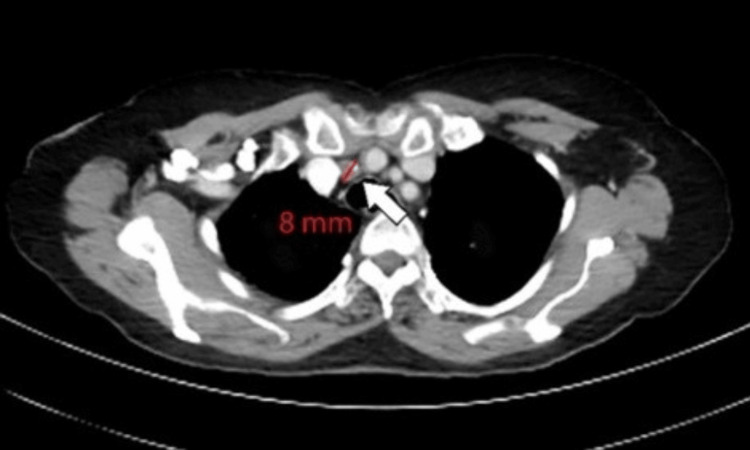
CT component showing a prominent pretracheal lymph node measuring 8 mm in dimension (arrow) CT: computed tomography

**Figure 6:**
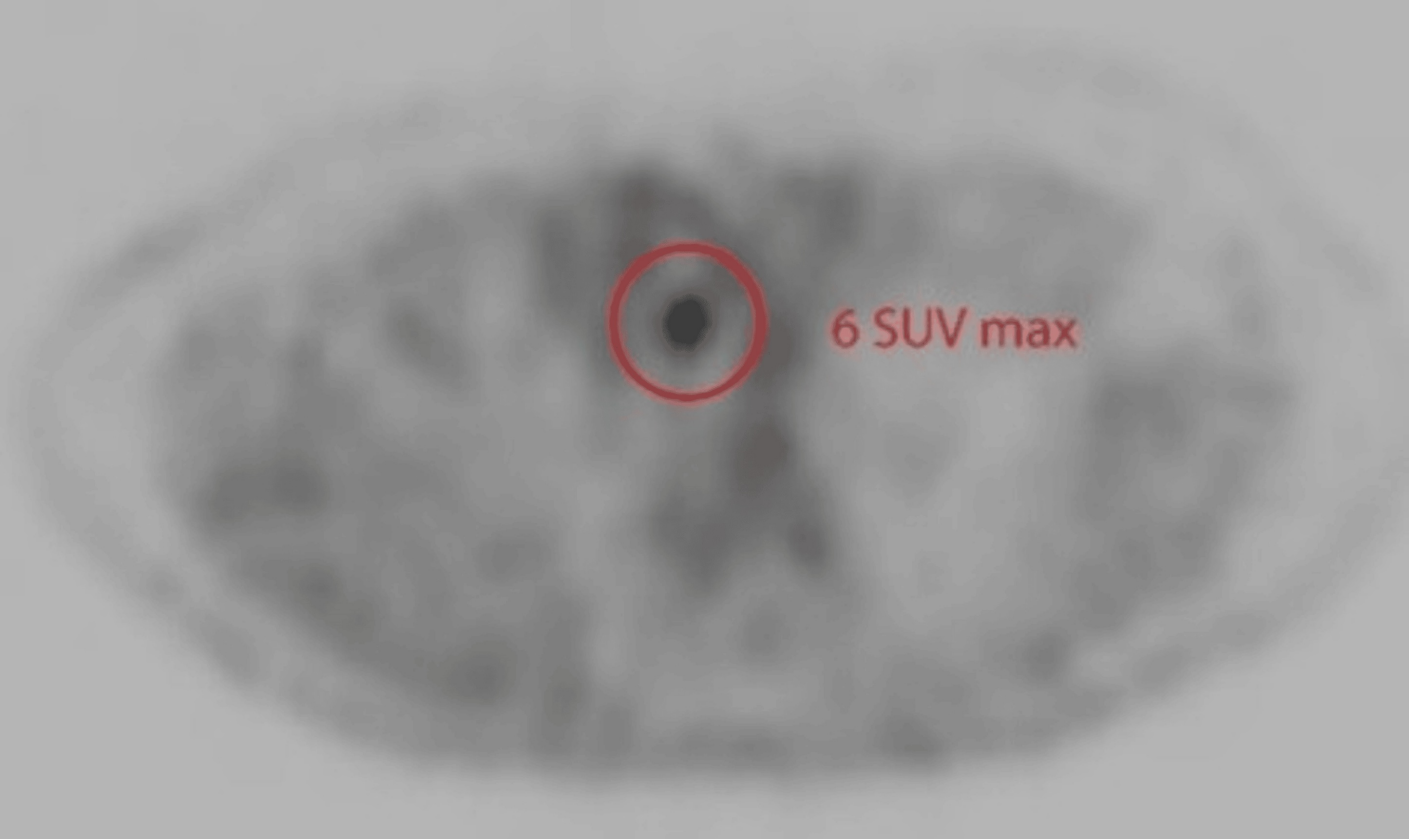
PET component showing increased metabolic activity of 6 SUV (red circle) PET: positron emission tomography; SUV: standardized uptake value

A 62-year-old man was initially diagnosed with squamous cell carcinoma of the lung. FDG-PET/CT scan was done for staging as seen in Figures 5-6. The CT component showed a prominent pretracheal lymph node measuring 8 mm in dimension (Figure 5). It was noted that this lymph node had an increased metabolic activity of 6 SUV raising concern (Figure 6). PET/CT decision was consistent with it being malignant and also turned out to be malignant on EBUS-guided fine-needle aspiration cytology (FNAC).

**Figure 7:**
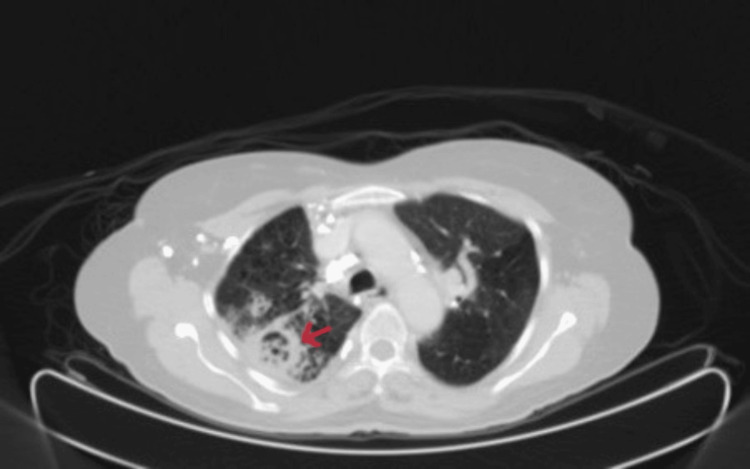
CT component in lung window, showing a cavitary lesion, seen in the posterior segment of the right upper lobe (arrow) CT: computed tomography

The CT component of the patient mentioned in the preceding paragraph, a 62-year-old man with the initial diagnosis of squamous cell carcinoma of the lung, in the lung window with a cavitary lesion seen in the posterior segment of the right upper lobe (Figure 7).

**Figure 8:**
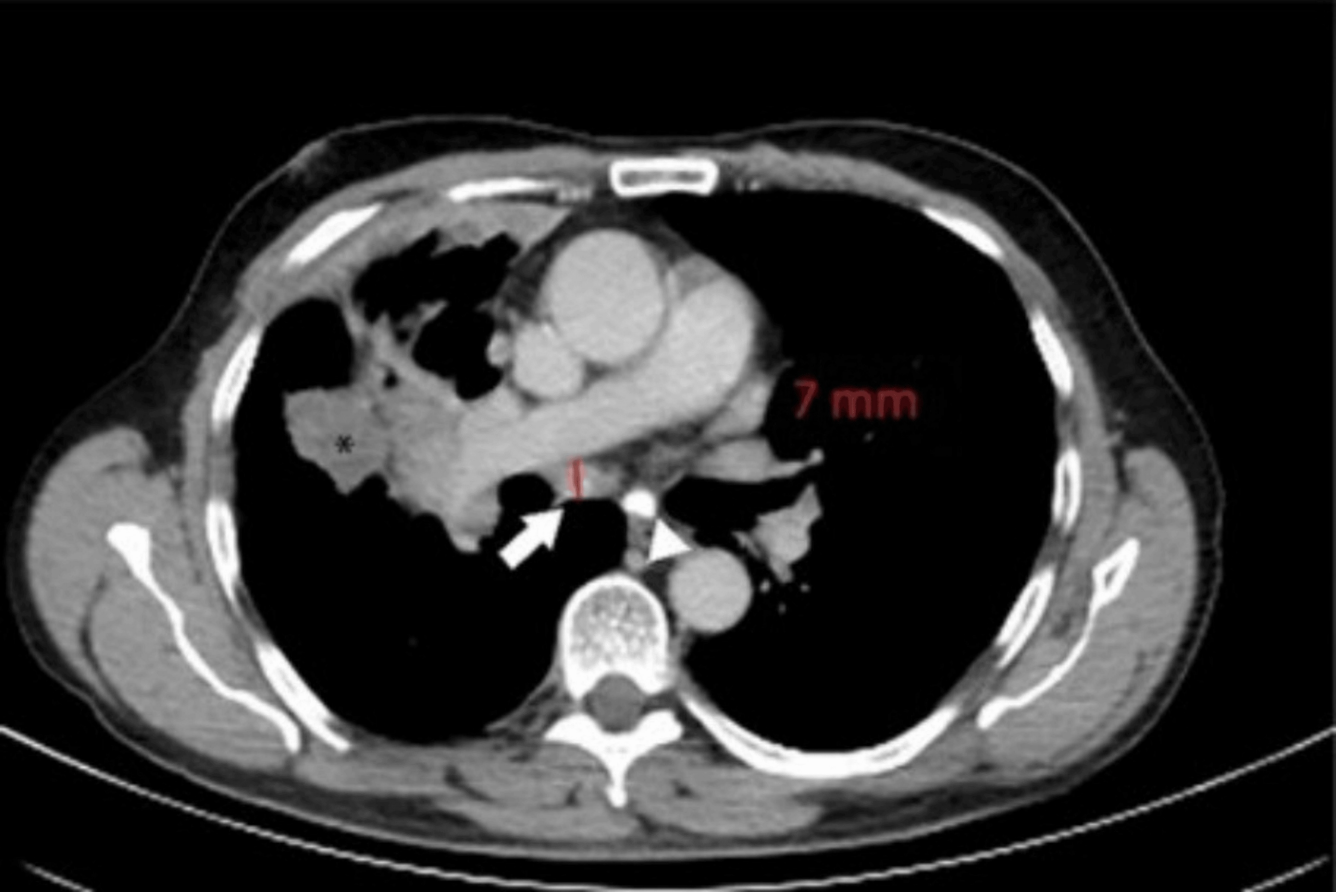
CT component showing a large heterogeneous solid mass in the right lung perihilar location, a prominent subcarinal node measuring 7 mm in short-axis dimension (arrow), and nodal calcifications in another subcarinal lymph node (arrowhead) CT: computed tomography

**Figure 9:**
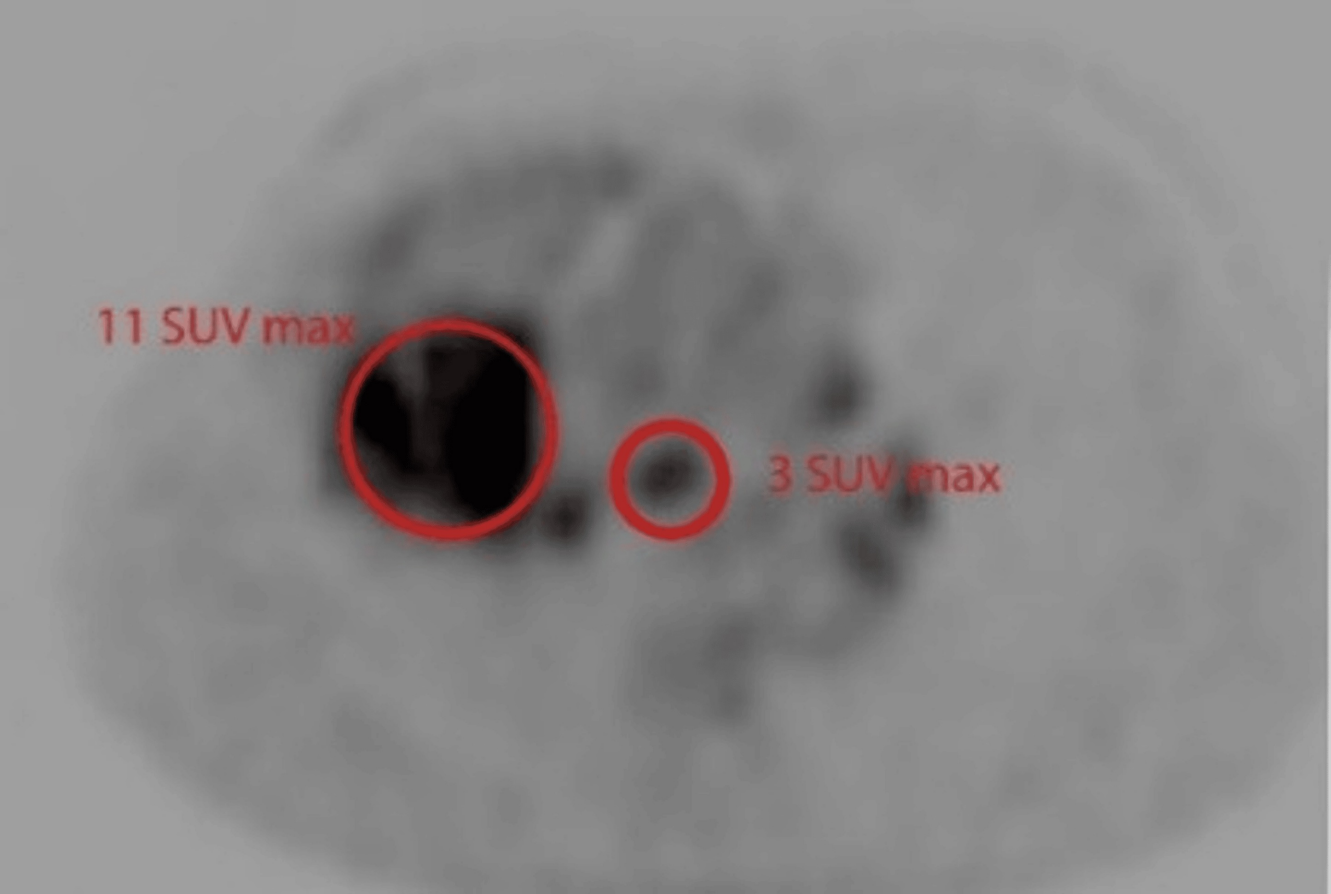
PET component, axial slice, from the same level, showing high metabolic activity in the right perihilar mass as well as in the mediastinal lymph node, with the mass itself showing metabolic activity of 11 SUV, while the maximum SUV of the subcarinal lymph node measures up to 3, indicating a lesser probability of metastasis PET: positron emission tomography; SUV: standardized uptake value

A 70-year-old male patient was initially diagnosed with lung adenocarcinoma and had a history of old pulmonary tuberculosis. FDG-PET/CT scan done for staging purposes showed a large heterogenous solid mass in the right lung perihilar location (Figure 8). A prominent subcarinal node was noted measuring 7 mm in the short axis dimension (Figure 9) with metabolic activity of 3 SUV. Nodal calcifications, especially in another subcarinal lymph node, were also noted (Figures 8-9). It was interpreted as mediastinal lymphadenopathy related to background tuberculosis rather than metastasis and was further confirmed on EBUS-guided FNAC.

## Discussion

Lung cancer is among the top causes of mortality due to malignancy globally, with lower survival rates and impacting males more than females [12,13]. However, this gap is seen to be closing due to a shift in the roles that women of this day and age play in our society. This is due to a handful of reasons, to name a few, presentation of men with more advanced stages of lung CA, stronger immune responses in women which lead to better response to treatments in women, and occupational exposures, that are observed to be higher in men. Additionally, cultural influences, like in the population our study was conducted in, often lead to higher smoking rates in men, another fact that contributes to the observed gender disparity in lung CA [13].

Approximately 75% of patients present with either locally advanced disease or one in which the disease process has metastasized, leaving the option of palliative treatment mostly. A point worth considering is that metastasis that has spread to the lymph nodes frequently appears in early-stage lung cancer and plays a crucial role in disease staging. Aside from malignancy, mediastinal lymph node enlargement can also be encountered in various other clinical conditions, such as inflammatory or infectious processes. CT scans and MRI have been widely used for the early establishment of diagnosis and staging of lung CA. On the one hand, these modalities provide a detailed structure of the involved lymph node; on the other hand, these can easily overlook lymph nodes below 1 cm. Conventionally, lymph nodes below the size threshold of 1 cm are considered normal, assuming metastatic nodes are larger. The CT scan shows a very low sensitivity (57%) when it comes to detecting subcentimeter lymph nodes leading to high false negatives for metastatic subcentimeter nodal involvement. Furthermore, CT can detect tumors that may not cause symptoms or harm, leading to unnecessary treatment and associated costs [14]. Interestingly, smaller lymph nodes have shown an increased likelihood of metastatic involvement compared to larger nodes, rendering size, when taken independently, an unreliable indicator of lymph node metastasis [9,15].

PET imaging has become an emerging means to assess lymph node involvement in metastatic disease taking metabolic activity into account. FDG is a glucose analog that is utilized in FDG-PET scans to evaluate glucose metabolic rates. It has been noted that FDG has an affinity for reactive lesions as well, thereby making it rather less specific for malignancy [16]. The introduction of combined FDG-PET/CT aims to overcome the limitations of using CT or FDG-PET alone for identifying malignant lesions. This combined approach not only has demonstrated higher sensitivity and specificity compared to CT alone but has improved accuracy in detecting nodal involvement [17]. 

In this research, we have studied the role of integrating FDG-PET/CT scans in determining the benign or malignant nature of involved subcentimeter lymph nodes in CA lung patients. Our study included 42 lung CA patients with evidence of subcentimeter lymph nodes. The mean age was 68 years, and a male predilection was observed in all patients. On histological evaluation, around 60% of patients had adenocarcinoma, while the remaining 40% had squamous cell CA. It was observed that out of the total of 26 patients with malignant nodes on histopathology, 19 (73.1%) were identified as malignant by FDG-PET/CT, and one out of 16 benign lymph nodes turned out to be malignant.

Metabolic activity is measured in terms of SUV and serves as a means to quantify FDG uptake in integrated PET/CT. Both SUV mean and SUV max play a role in diagnosing as well as distinguishing between benign and malignant lesions. However, SUV max is favored due to its ability to significantly reduce interobserver variability, which is seen to drop from 35% to merely 3% when compared to SUV mean [18]. Based on this, and as indicated in a study conducted by Gambhir, using a cutoff SUV max value has promised more accuracy in differentiating between benign and malignant lesions [19]. Thus, in our study, SUV max was utilized for calculating metabolic activity.

In Knight et al.’s study, the cutoff value of SUV in order to differentiate between benign and malignant lesions was found to be 2.5 [20]. However, later on, a study done by Goo [21] proved that there is a need to increase the SUV max cutoff value due to significant overlapping and compromised distinction between benign and malignant lesions. In our study, at 2.5 SUV max cutoff value, a very low specificity (10%) was observed. It can be attributed to the increased prevalence of inflammatory disease and reactive nodes in our population. Our study supports the results of Goo and meta-analysis by Liu et al., and it was shown that the SUV cutoff value when increased from 2.5 to 5.5 resultantly raised the sensitivity and specificity rates to 65% and 91%, respectively, using the ROC curve. Similar results depicting a remarkable increase in sensitivity and specificity by increasing the cutoff value to 5.3 were observed in a meta-analysis done by Liu et al. in 2016 [21,22].

The optimal size cutoff was obtained to be 7.5 mm at which the highest specificity and sensitivity were seen. Most of the malignant nodes had sizes ranging from 7 to 9 mm, whereas benign nodes had relatively smaller sizes. In a meta-analysis conducted by Wang et al., it was revealed that, in addition to metabolic activity, several other factors can also predict nodal metastasis, with size emerging as another important determinant [23]. Aligning with the findings of our study, among subcentimeter lymph nodes, a significant proportion of metastatic nodes ranged in size from 7 to 9 mm. Likewise, research conducted by Zhou et al. has highlighted a notable correlation between lymph node size and metastasis [24].

Although both SUV max and size can independently direct on the presence of lymph node metastasis, combining both to detect malignant lymph nodes can enhance accuracy. As a diagnostic tool, FDG-PET/CT has shown high sensitivity of 95%, moderate specificity of 68%, positive predictive value of 73%, negative predictive value of 94%, and overall accuracy of 80.9%.

FDG-PET/CT as a combined modality is more sensitive and specific than CT scan alone as depicted in a study done by Yi et al. In their study, the CT scan detected sensitivity of lymph node metastasis was 81% and accuracy of 85%, while FDG-PET/CT exhibited both greater sensitivity of 96% and accuracy of 93%. Our study findings align closely with Yi et al., where FDG-PET/CT displayed a sensitivity of 95% [25]. Similarly, a meta-analysis by Birim et al. has shown that PET/CT is better than CECT scans. PET/CT has a sensitivity of 57%-68%, and CECT scans have a sensitivity of 79%-85%. Similarly, PET/CT has a specificity of 76%-82%, whereas CECT scans have a specificity of 87%-92% [26]. Sun et al. demonstrated a wider range of sensitivity (50%-95%) and specificity (82%-99%) on PET scans alone. Yet, when FDG-PET/CT is utilized, it has shown more favorable and accurate staging compared to conventional imaging methods [27].

A study by Pfannenberg et al. in 2007 on staging NSCLC patients found FDG-PET/CT to have lower sensitivity (68%) but higher specificity (90%) for detecting nodal metastasis and achieving precise staging, contrasting our study where sensitivity was higher at 95% but specificity lower at 68% [28]. Conversely, a study conducted by Behzadi et al., discussing the detection of lymph node metastasis in lung cancer, revealed that FDG-PET/CT has high sensitivity but low specificity, with an accuracy of 90%, which falls close to the accuracy of 80.9% found in our study [29].

Size and metabolic activity are used as predictors of nodal metastasis in subcentimeter lymph nodes. Early staging of NSCLC can be done using FDG-PET/CT as this modality provides high specificity as well as a greater positive predictive value in detecting nodal metastasis. For accurate staging and a better treatment approach, there is a potential for FDG-PET/CT to replace conventional imaging modalities like CT and MRI especially when these are used independently. Based on the results of our study, we propose further research on a bigger and more diverse population in order to move toward considering FDG-PET/CT as the initial imaging modality, before a CT scan, due to its greater sensitivity in detecting subcentimeter lymph node involvement in metastatic disease. This adjustment in the diagnostic approach could enhance early detection and improve treatment planning. There are approximately 19,000 new cases and 14,000 deaths owing to lung cancer annually in Pakistan [30]. This study marks an important leap toward early detection which is crucial due to its time-sensitive nature for patients with subcentimeter metastasis of lung CA and subsequent management of the disease, within the country.

It is equally important to address notable limitations like increased occurrence of false-positive rates. Moreover, with the setting of this study being Pakistan, challenges like limited access to advanced imaging and trained staff, high cost of this procedure, variable access to healthcare facilities, and difficulty in diagnosing diseases like tuberculosis being prevalent, were faced. However, our study's outcomes in Pakistan can be optimized by taking some steps like possible future collaboration with international institutions for access to advance technology, specialized diagnostic training for more local healthcare professionals, ensuring the implementation of strict quality control measures, and educating the population. These steps are pivotal in overcoming the abovementioned challenges and ensuring our study's success in contributing valuable insights to lung cancer management in Pakistan.

## Conclusions

FDG-PET/CT is a noninvasive imaging modality that can be used in the detection of subcentimeter metastatic lymph node involvement in lung CA. It has high sensitivity and accuracy but a low specificity rate to detect nodal involvement in lung CA patients. The high false-positive rates are mainly due to the increased prevalence of endemic lung infectious diseases.
